# Translation, cross-cultural adaptation and reliability of the German version of the migraine disability assessment (MIDAS) questionnaire

**DOI:** 10.1186/s12955-018-0871-5

**Published:** 2018-03-09

**Authors:** Thomas Benz, Susanne Lehmann, Andreas R. Gantenbein, Peter S. Sandor, Walter F. Stewart, Achim Elfering, André G. Aeschlimann, Felix Angst

**Affiliations:** 1 Rehabilitation Clinic “RehaClinic”, Bad Zurzach, Switzerland; 2 Institute of Psychology, University of Bern, Bern, Switzerland; 3 University of Zurich, Zürich, Switzerland; 4 Research and Development, Sutter Health, Concord, California, USA

**Keywords:** MIDAS, German, Cross-cultural adaptation, Reliability

## Abstract

**Background:**

The Migraine Disability Assessment (MIDAS) is a brief questionnaire and measures headache-related disability. This study aimed to translate and cross-culturally adapt the original English version of the MIDAS to German and to test its reliability.

**Methods:**

The standardized translation process followed international guidelines. The pre-final version was tested for clarity and comprehensibility by 34 headache sufferers. Test-retest reliability of the final version was quantified by 36 headache patients completing the MIDAS twice with an interval of 48 h. Reliability was determined by intraclass correlation coefficients and internal consistency by Cronbach’s α.

**Results:**

All steps of the translation process were followed, documented and approved by the developer of the MIDAS. The expert committee discussed in detail the complex phrasing of the questions that refer to one to another, especially exclusion of headache-days from one item to the next. The German version contains more active verb sentences and prefers the perfect to the imperfect tense. The MIDAS scales intraclass correlation coefficients ranged from 0.884 to 0.994 and was 0.991 (95% CI: 0.982–0.995) for the MIDAS total score. Cronbach’s α for the MIDAS as a whole was 0.69 at test and 0.67 at retest.

**Conclusions:**

The translation process was challenged by the comprehensibility of the questionnaire. The German version of the MIDAS is a highly reliable instrument for assessing headache related disability with moderate internal consistency. Provided validity testing of the German MIDAS is successful, it can be recommended for use in clinical practice as well as in research.

**Electronic supplementary material:**

The online version of this article (10.1186/s12955-018-0871-5) contains supplementary material, which is available to authorized users.

## Background

In the adult population, headache disorders have been reported to be among the 10 most disabling conditions on the World Health Organization’s ranking of causes of disability [1]. The total annual cost of headache in adults aged 18–65 years in the EU was estimated at 173 billion Euros, reflecting the high individual and societal impact [2]. Headache disorders have a very high impact on patients and lead to limitations in daily activities like work or school, household activities, and social and leisure activities [3]. Loss of days for work, housework or social activities due to migraine was more than 10% in 28.0% of female and 17.7% of male patients [3].

There are several instruments that measure the disease-specific and health-related disability and its impact on the quality of life of headache sufferers. The Migraine Disability Assessment (MIDAS) questionnaire was originally developed to assess the migraine-related disability of English speaking patients [4]. This tool is one of the most widely used questionnaires and measures impact of headache on the functioning at work, in school and in social activities by assessing lost days in the past 3 months [5]. MIDAS questions are not specific for a type of headache, but ask about headache in general. This means that the MIDAS can be used for any types of headaches including acute or chronic migraine, tension-type headache, episodic headache, and medication-overuse headache.

It is a short, easy to use and score questionnaire with 7 items. The first 5 questions assess completely lost days and the days with a reduced productivity of at least 50%, which add up to give the MIDAS total score. The frequency and intensity of the headaches over the past 3 months are assessed by the last 2 questions. The grading system of the MIDAS categorises the MIDAS total score into Grade I to IV, from minimal or infrequent disability with a score of 0–5 (Grade I) to severe disability with a score of 21 or more (Grade IV).

The MIDAS questionnaire is available for use free of charge and can be used in clinical practice as well as in research. It was designed to improve patient-doctor communication, to measure headache-related disability, and to stratify patients by treatment needs [6]. It also meets physicians’ conceptions of important clinical criteria and seems, therefore, suitable for use in clinical practice [7–9]. All outcome domains of the MIDAS were considered important to people with chronic pain and are among the outcome domains recommended by the Initiative on Methods, Measurement, and Pain Assessment (IMMPACT) [10].

Reliability and validity of the MIDAS questionnaire have been tested extensively in numerous studies in various countries and in different languages [4, 7, 11–20]. A German version of the MIDAS was published by Agosti et al. in 2008 [21]. This version was linguistically validated by a second translation by an independent and qualified translator. However, the steps of forward- and backward-translation proposed by the cross-cultural adaptation process were not completely followed [22]. Reliability and validity of this version was not tested. So far, no specific self-assessment instrument in German measuring headache-related disability in patients with migraine has been translated in accordance with the above-mentioned international guidelines [22].

The first goal of this study was to translate and cross-culturally adapt the original English version into German according to the standardized, well-established procedure [22]. The second aim of the study was to describe the metric properties and to quantify test-retest reliability. Further, the full length German version of the MIDAS questionnaire should be published for free use.

## Methods

This study was conducted at the rehabilitation clinic “RehaClinic” in Bad Zurzach, Switzerland. Two samples were recruited, one to test the pre-final version of the German MIDAS and one for test-retest reliability. Written informed consent was obtained from all participants in this study. The study protocol was approved by the Local Ethic Commission (Health Department in Aarau, Switzerland, EK AG 2008/026).

### Translation and cross-cultural adaptation

Translation and cross-cultural adaptation was done according to the international guidelines of the American Association of Orthopedic Surgeons (AAOS) Outcome Committee and was preliminary discussed and approved by the developer of the MIDAS, Walter. F. Stewart [22]. This procedure is based on the original guideline of Guillemin et al. 1993 and has been further developed and illustrated by Beaton et al. and other authors [22–25]. The translational process described in all those studies emphasizes the importance of the translation process and translation quality in order to maximize attainment of equivalence. The components of the process are principally equal in all those studies and are considered to be exemplary in the fundamental textbook of Streiner et al. [26]. It consists of the following 6 stages:

1. Forward translation
  Two bilingual (fluent in written and spoken German and English) German native speakers independently translated the MIDAS from English to German. One translator was informed about the intention of the questionnaire and had a medical background. The second translator did not have any medical background and was without any prior knowledge of the purpose and use of the MIDAS. Both independently delivered a written report.
2. Synthesis of the 2 first German versions
  The two written reports were synthesized to one German version by consensus by the two translators and recorded by the study nurse.
3. Back translation of the synthesis
  The synthesis of the two translations to German was then independently translated back to English by 2 persons with English as a native language and excellent German language and culture knowledge. The 2 translators both had a different original cultural background, one from New Zealand, one from England.
4. Expert committee review to achieve the pre-final German version
  The expert committee consisted of all the translators, 1 general practitioner and epidemiologist, 1 physiotherapist and 1 research assistant. This committee reviewed the original version, German versions and the back-translations. By consensus, the expert committee formulated the pre-final version by discussing the semantic, idiomatic, experiential and conceptual equivalence, made the necessary changes and documented the adaptations in a written report.
5. Testing of the pre-final version and adaptation to the final German version
  The pre-final version was tested on *n* = 34 persons who all suffered from headache, recruited from December 2014 till March 2015. The majority (*n* = 26) was included by convenience sampling of consecutively admitted patients to the Pain Center of the rehabilitation clinic “RehaClinic” in Bad Zurzach, Switzerland. It consists of three specific, in-house, multidisciplinary rehabilitation programs: for medication overuse headache, for whiplash associated disorders, for chronic musculoskeletal pain disorders. Further, 4 ambulatory patients from general practice and 2 employees completed the questionnaire. This sample also covered 2 twelve-year-old teenagers with headache representing persons with limited education as recommended [22]. All subjects rated clarity and comprehensiveness of the title, the instructions and the questions on a scale from 0 (=not comprehensible at all) to 10 (=easily comprehensible). Furthermore, free comments about what he or she thought was meant by each questionnaire item and the chosen response could be added [22]. Further, time needed to fill out the questionnaire was recorded. Based on the comments of the pre-test, final adaptations were made by the expert committee [27].
6. Submission and Appraisal of all written reports by the developer
  Each process was documented in a written report which was approved by the developer of the questionnaire, W.F. Stewart.

### Test-retest reliability testing

Test-retest reliability testing was started after completion of stage 6 in March 2015 and lasted till September 2015. Consecutive convenience sampling in the Pain Center of the rehabilitation clinic “RehaClinic” in Bad Zurzach, Switzerland was performed to obtain a new sample different from the sample for testing the pre-final version (*n* = 36). Of those, *n* = 33 were recruited at the Pain Center and n = 3 from general practice, all suffering from chronic episodic headache. Sample size was pretermined. To detect an intraclass correlation coefficient (ICC) = 0.80 with a power = 80% and type I error = 0.05 a sample size of n = 36 patients was necessary [28].

All patients were tested twice with a time interval of 48 to 60 h between completed questionnaires. We assumed that 48 h was enough time for patients not to recall the results of the first completed questionnaire. By that, the 2 time windows of 90 days recall were highly congruent. For reliability testing, the period of 2 days was considered to be equivalent to that of 2 weeks [29]. The first questionnaire was given to the in-house patients on Friday and patients were asked to fill out the questionnaire on Friday evening after the last medical treatment. The second questionnaire was filled out on Monday morning before the first treatment. The time period in between was considered as stable because no medical treatment was administered.

### Analysis

For the pre-test results of clarity and comprehensibility the mean averages of the different components and the time needed to complete the questionnaire were calculated. Based on these average scores and the test persons’ comments, the pre-final version of the questionnaire was adapted.

Test-retest reliability of the final version was quantified by the ICC. The ICC quantifies the extent to which the same test results are obtained for repeated measurements when no change of results is expected between the two assessments. We calculated the ICC for each question on the MIDAS (question 1–5 and additional questions A + B) and the total MIDAS score. For better classification, the MIDAS total score was transformed into a scale from 0 (no days) to 100 (maximum of 276 days = worst score).

The ICC score ranges from 0.00 indicating no reliability to 1.00 indicating perfect reliability. An ICC ≥ 0.80 reflects high test-retest reliability and means that 64% (0.80 squared) of the variance of the test scores are explained by the retest scores.

The internal consistency of the German version of the MIDAS total score of items 1–5 was determined by Cronbach’s alpha (α) at test. A Cronbach’s α of 0.7 is deemed acceptable and of value of 0.8 is deemed excellent internal consistency. At test assessment, the floor effect was quantified as the percentage of patients who achieved the worst possible score and the ceiling effect as the percentage achieving the best possible score.

The extent of agreement between the first and the second measurement was examined with a Bland-Altman plot [30]. This method plots the difference between the pairs of the two measurements against the mean of each pair of measurement. The limits of agreement were calculated as the mean difference +/− twice the standard deviation [26].

All analyses were performed using the statistical software package IBM SPSS 23.0 for Windows® (SPSS Inc., Chicago, IL, USA).

## Results

### Translation and cross-cultural adaptation process

The procedure of stages I – VI as described by Beaton et al. [22] was planned carefully and realised as required without deviation from the protocol.

In the instructions for the original questionnaire, the following phrase was given in brackets: “Please refer to the calendar below, if necessary.” A calendar was not included in the original version. Therefore, this phrase was excluded in the final version by the expert committee at stage IV and with the permission of the author of the original questionnaire (stage VI).

The forward translation resulted in activities which were expressed in the imperfect (past tense) in German (stages I – II). Back-translation led to perfect tense in English in some items (stage III). The expert committee decided to use the German perfect tense consistently across all items to reflect activities which have happened in the last 3 months up to present (stage IV). Perfect tense and imperfect tense are almost synonymously used in Germany, but in Swiss German only perfect tense exists. To clarify, the MIDAS was translated into “German” German, which is the written and spoken language in Germany and the written language in Switzerland.

All members of the expert committee rated the instruction “do not include days you counted in question 1 where you missed work or school” (item 2) as very difficult to understand. It was considered very likely that mistakes in counting the number of days for items 2 and item 4 might occur because of it. However, the instruction was translated word for word to stay consistent with the original version.

### Pre-test (stage V)

Clarity and comprehensibility were rated by the 34 persons suffering from headache with an average score of 3.76 for the title, 8.74 for the instructions and 8.5 for the questions of the MIDAS. For all 34 questionnaires with a total of 238 items, there were 9 missing items (3.8%). The average time needed to complete the questionnaire was 3.62 min. The 2 teenagers did not have any problems understanding and filling out the questionnaire.

Many respondents stated under comments that sentences had to be read at least twice to understand the exact content. For those participants who had headaches during the rating procedure, concentration was reported to be difficult. Specifically, some patients were challenged in recalling the exact number of days in the last 3 months with limitations due to their headaches. Six of the 34 persons reported difficulties in remembering the number of headache days of the last 3 months. Seven of the 34 participants found it very difficult to subtract the number of headache days of the previous item in items 2 and 4. Patients not working due to unemployment, retirement or a sick certificate found the questionnaire to be inapplicable to their situation. However, these difficulties did not lead to adaptations of the pre-final version to keep congruence with the original version.

Based on the results of the pre-test, we concluded that the German version of the MIDAS questionnaire is a comprehensible questionnaire which can be filled out in less than 4 min. Adaptation of the title was needed to make it more comprehensible. The title in the pre-final version “Der MIDAS Fragebogen” (translated in English: “the MIDAS questionnaire”) was changed to “Der MIDAS Fragebogen (Migraine Disability Assessment)” and completed with an additional explanation of the goal of this questionnaire: “Fragebogen zur Erfassung der funktionellen Einschränkung durch Kopfschmerzen, insbesondere Migräne” (translated in English: “questionnaire to assess disability related to headaches, especially migraine”). The Additional file 1 shows the final German version of the MIDAS questionnaire.

### Reliability testing

Of the total sample (*n* = 36), mean age was 43.8 (standard deviation (sd) = 14.5), 27 (75%) patients participated in the medication overuse headache program, and 30 (83%) were female.

At test (first administration of the MIDAS), the mean number of days with disability due to headache ranged from 14.5–22.0 across the items 1–5 (Table 1). The average number of days with headache (item A) was reported 61.4 days with large variation (sd = 27.3). Pain severity (item B) was on average 5.86 (sd = 5.87, 10 = worst pain). The total MIDAS score for items 1–5 averaged to 68.1 (100 = no disability due to headache).

**Table 1:** Descriptive (at test) and reliability data for the German MIDAS (*n* = 36)

Item	Wording of the original MIDAS [4]	Minimum	Maximum	Mean	SD	ICC	95% CI
1	On how many days in the last 3 months did you miss work or school because of your headaches?	0 (41.7%)	92 (2.8%)	14.47	26.75	0.988	0.976–0.994
2	How many days in the last 3 months was your productivity at work or school reduced by half or more because of your headaches? (Do not include days you counted in question 1 where you missed work or school.)	0 (33.3%)	90 (0.0%)	21.00	27.37	0.962	0.926–0.980
3	On how many days in the last 3 months did you not do household work (such as housework, home repairs and maintenance, shopping, caring for children and relatives) because of your headaches?	0 (19.4%)	90 (0.0%)	17.17	21.27	0.989	0.979–0.994
4	How many days in the last 3 months was your productivity in household work reduced by half or more because of your headaches? (Do not include days you counted in question 3 where you did not do household work.)	0 (19.4%)	90 (0.0%)	20.69	23.26	0.896	0.806–0.946
5	On how many days in the last 3 months did you miss family, social or leisure activities because of your headaches?	0 (8.3%)	90 (0.0%)	22.03	26.87	0.994	0.988–0.997
A	On how many days in the last 3 months did you have a headache? (If a headache lasted more than 1 day, count each day.)	5 (0.0%)	92 (2.8%)	61.44	27.27	0.920	0.849–0.958
B	On a scale of 0–10, on average how painful were these headaches? (where 0 = no pain at all, and 10 = pain as bad as it can be.)	1 (0.0%)	10 (2.8%)	5.86	1.57	0.884	0.785–0.939
	Total Score MIDAS Items 1–5 (276 = worst)	0 (5.6%)	235 (0.0%)	87.97	70.47	0.991	0.982–0.995
	Total Score MIDAS Items 1–5 (0 = worst, 100 = best)	14.86 (0.0%)	100 (5.6%)	68.13	25.53	0.991	0.982–0.995

*Legend*: The percentages in parentheses in the Minimum and Maximum column indicate the frequencies of floor and ceiling values: minimum: percentage with 0 = ceiling; maximum: percentage with worst possible score = floor; for items 1–5 (92 = worst), A (92 = worst), B (10 = worst) and MIDAS total score (276 = worst). For MIDAS total score (100 = best) vice versa

*MIDAS* Migraine Disability Assessment, *SD* Standard Deviation, *ICC* Intraclass correlation coefficient, 95% CI: 95% confidence interval for the ICCs

Frequencies of floor scores (worst possible scores, Table 1) were low: 1 patient on item 1 and the same patient in question A (number of headache days per 3. months). Further, one rated worst pain on the Numeric Rating Scale (NRS) (Item B). Ceiling phenomena were very frequent in items 1–5, especially in item 1 (41.7% reported 0 days of missing work or school because of your headaches in the last 3 months) and least in item 5 (8.3% reported 0 days of missing family, social or leisure activities because of your headaches in the last 3 months). Ceiling on the total MIDAS score was rated by 2 patients (0 days missing/reduced productivity due to the headaches in items 1–5). There was no ceiling on item As and B.

All 7 items as well as the MIDAS total score showed an ICC equal to or greater than 0.884 (Table 1). Item B, the average of pain intensity, was lowest with an ICC of 0.884 whereas item 5, which asked for the lost days in family, social or leisure activities was 0.994. The MIDAS total score showed a high ICC of 0.991. The internal consistency of items 1–5 at test was Cronbach’s α = 0.690 (95% CI 0.495–0.825) and 0.670 (95% CI 0.463–0.814) at retest.

**Fig. 1:**
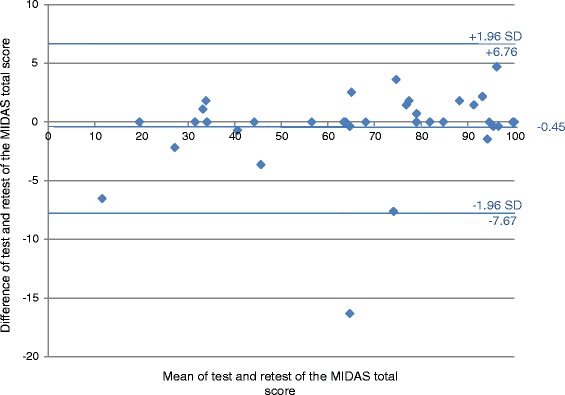
Bland-Altman plot of the MIDAS total score

The Bland-Altman plot (Fig. 1) showed that the mean difference in the total MIDAS score between the two measurements was − 0.45 and the 95% limits of agreement were 6.76 and − 7.67. Differences between the first and second measurements were within the limits of agreement (mean +/− twice the standard deviation). There was, however, one clear outlier. The plot indicated that there was agreement between the measurements and that there was no relationship between the differences and the MIDAS total score. The difference between the two measurements did not get larger or smaller with an increased average.

## Discussion

We translated and cross-culturally adapted the original English version of the MIDAS into German and tested its test-retest-reliability. As a result, the German MIDAS seems comprehensible and linguistically very close to the original version and patient responses were highly reliable.

Several problems emerged with the assessment period and the content of the MIDAS questions based on the standardized 6 stages of translation and cross-cultural adaptation. Especially in stage V: cross-sectional testing of the pre-final version, many persons reported that it was difficult to accurately recall the number of headache days within the last 3 months and that the content of some items is difficult to understand. For example, problems caused items 2 and 4 where the number of headache days assessed by the previous item has to be subtracted. These difficulties of comprehensibility are aggravated if the participants have a headache at the time and their concentration is affected. These problems led to lengthy discussions in the consensus conference team (stage IV). However, the phrasing of the final German version is very close to the original version, which was supported by the agreement of the developer W.F. Stewart. After the title, the additional phrase highlights that the questionnaire asks about limitations due to headache. For better comprehensibility, the English imperfect/past tense was changed to the German perfect tense throughout the questionnaire.

In the development of the original MIDAS, it turned out that a 90 day recall period did offer a more reliable assessment of patient’s experience than a 45 day recall period and that the corresponding numbers of days of disability due to headache (items 1–5) were similar to those assessed by a daily headache diary [12].

In reliability testing on 36 persons suffering from headache, items 1 and 2 showed high and items 3 and 4 moderate ceiling effects, whereas item 5 and the total score had low ceiling effects. Floor effects were very low or absent. Although pain levels were moderately high (on average 5.86) and the mean number of headache days was two of three months (item A), a high proportion of the participants did not feel disabled to perform work (items 1 and 2). In the validity study of the original MIDAS it was shown that affected persons firstly reduce household chores, social, and leisure activities before they miss work or school [12].

The test-retest (2 to 2.5 days later) ICC’s of the 7 items were very high and ranged from 0.884 (item B: pain NRS) to 0.989 (item 3: disability in household work). All ICC’s were statistically significant at type I error = 0.05 and a power of 0.80 (type II error = 0.20). The total MIDAS score composed of the sum of items 1 to 5 was very highly reliable (ICC = 0.991), but the 5 items showed moderate internal consistency (Cronbach’s α = 0.690). This means that the content of the five items reflects some divergence in the constructs. For example, retired and unemployed persons have difficulties rating disability at work or school (items 1 and 2), in contrast to disability concerning household work (item 3 and 4), or disability for social activities (item 5). The original MIDAS aimed to cover all those 3 constructs separately and the relatively low Cronbach’s α reflects that this strategy was successful [4].

Our high test-retest reliability stays in contrast with the relatively lower test-retest Spearman correlation of between 0.46 and 0.78 for items 1 to 5 of the original MIDAS version in migraine headache sufferers [7]. However, the original MIDAS showed a good correlation with physicians’ assessment and a diary-based measurement of disability [12].

The original MIDAS has been translated into several languages and the reliability of the Chinese [13], French [14], Hindi [15], Japanese [16], Malay [17], Persian [18], Thai [19], and Turkish [20] versions has been tested. The test-retest Pearson or Spearman correlation coefficients in the different versions ranged from 0.84 to 0.87 for single items of the MIDAS and from 0.65 to 0.94 for the MIDAS total score. Cronbach’s α for internal consistency ranged from 0.65 to 0.84. The results of the German version of the MIDAS in this study are comparable to those reported for the original English as well as the various translations of the MIDAS.

The standardized process of translation and cross-cultural adaptation was strictly followed. This is a strength of this study. The process was characterized by intensive discussions about the equivalence of the content and the precise phrasing of the pre-final version in stage IV. Minor adaptations in the title of the questionnaire improved the comprehensibility. Another strength of this study is that the number of patients in the pretest was reached as proposed by Beaton et al. [22]. The sample size for reliability testing was predefined by sample size calculation. Test and retest assessments were performed in a period without therapy where the state of the patient was assumed to be stable.

The selection of patients at an inpatient rehabilitation clinic from those participating in a program for headache disorders and after withdrawal from medication overuse may limit the generalizability of the results. Validity testing of the German MIDAS will complete the psychometric properties of the instrument in future.

## Conclusions

The German version of the MIDAS presented in this study is a short, 7 item condition-specific questionnaire assessing disability due to migraine and headache, the number of headache days in the last 3 months and pain intensity (NRS 0–10). Although the phrasing of the questionnaire is challenging, it is a practical instrument for an assessment of headache related disability, which in general takes less than 5 min to fill out and can be obtained free of charge. The translation process was challenged by the comprehensibility of the questionnaire. The German version of the MIDAS is a highly reliable instrument for assessing headache related disability with a moderate internal consistency. Provided validity testing is successful, the MIDAS can be recommended for use in clinical practice as well as in research.

## Additional file

Additional file 1: German version of the MIDAS questionnaire (final version). Final German version of the MIDAS questionnaire. (DOCX 14 kb)

## Abbreviatons

CI: Confidence interval
ICC: Intraclass correlation coefficient
MIDAS: Migraine disability assessment score
NRS: numeric rating scale
SD: standard deviation
