# Supplementary material for: Translation, cross-cultural adaptation and reliability of the German version of the migraine disability assessment (MIDAS) questionnaire
Source: Health Qual Life Outcomes. 2018 Mar 9;16:42. doi: 10.1186/s12955-018-0871-5 (PMC5845367; doi:10.1186/s12955-018-0871-5)
Supplement: Supplementary file 1 — German version of the MIDAS questionnaire (final version). Final German version of the MIDAS questionnaire. (DOCX 14 kb) [file 12955_2018_871_MOESM1_ESM.docx]

**Additional file 1**

German version of the MIDAS questionnaire (final version)

**Der MIDAS Fragebogen (*MI*graine *D*isability *AS*sessment)**

| Fragebogen zur Erfassung der funktionellen Einschränkungen durch Kopfschmerzen, ins-besondere Migräne. | | | | |
| --- | --- | --- | --- | --- |
| **Anleitung:** Bitte beantworten Sie die folgenden Fragen über ALLE Kopfschmerzen, die Sie in den letzten 3 Monaten hatten. Schreiben Sie Ihre Antwort in das Feld neben jeder Frage. Schreiben Sie eine Null, wenn Sie die Tätigkeit/Aktivität in den letzten 3 Monaten nicht gemacht haben. | | | | |
| **1** | An wie vielen Tagen haben Sie in den letzten 3 Monaten wegen Ihren Kopfschmerzen an der Arbeit oder in der Schule gefehlt? | |  | Tage |
| **2** | An wie vielen Tagen haben Ihre Kopfschmerzen in den letzten 3 Monaten Ihre Leistungsfähigkeit bei der Arbeit oder in der Schule um die Hälfte oder mehr reduziert? (Schliessen Sie die Tage von Frage 1 nicht mit ein, an denen Sie an der Arbeit oder in der Schule gefehlt haben.) | |  | Tage |
| **3** | An wie vielen Tagen haben Sie in den letzten 3 Monaten wegen Ihren Kopfschmerzen keine Arbeiten im Haushalt gemacht? | |  | Tage |
| **4** | An wie vielen Tagen haben Ihre Kopfschmerzen in den letzten 3 Monaten Ihre Leistungsfähigkeit bei Arbeiten im Haushalt um die Hälfte oder mehr reduziert? (Schliessen Sie die Tage von Frage 3 nicht mit ein, an denen Sie keine Arbeiten im Haushalt gemacht haben.) | |  | Tage |
| **5** | An wie vielen Tagen haben Sie in den letzten 3 Monaten wegen Ihren Kopfschmerzen familiäre oder soziale Aktivitäten oder andere Aktivitäten ausserhalb der Arbeit verpasst? | |  | Tage |
|  |  | |  |  |
| **A** | An wie vielen Tagen hatten Sie in den letzten 3 Monaten Kopfschmerzen? (Falls die Kopfschmerzen mehr als einen Tag dauerten, zählen Sie jeden Tag.) | |  | Tage |
|  |  | |  |  |
| **B** | Wie schmerzhaft waren diese Kopfschmerzen im Durchschnitt auf einer Skala von 0 bis 10? (0= überhaupt kein Schmerz und 10= Schmerz, so schlimm wie er sein kann) | \| ○ \| ○ \| ○ \| ○ \| ○ \| ○ \| ○ \| ○ \| ○ \| ○ \| ○ \| \| --- \| --- \| --- \| --- \| --- \| --- \| --- \| --- \| --- \| --- \| --- \| \| 0 \| 1 \| 2 \| 3 \| 4 \| 5 \| 6 \| 7 \| 8 \| 9 \| 10 \| | | |
